# Magnetic Polyamide Nanocomposites for the Microextraction of Benzophenones from Water Samples

**DOI:** 10.3390/molecules24050953

**Published:** 2019-03-08

**Authors:** Hoda Ghambari, Emilia M. Reyes-Gallardo, Rafael Lucena, Mohammad Saraji, Soledad Cárdenas

**Affiliations:** 1 Department of Chemistry, Isfahan University of Technology, Isfahan 84156-83111, Iran; ghambari_h@yahoo.com (H.G.); saraji@cc.iut.ac.ir (M.S.); 2 Departamento de Química Analítica, Instituto Universitario de Investigación en Química Fina y Nanoquímica IUNAN, Universidad de Córdoba, Campus de Rabanales, Edificio Marie Curie, E-14071 Córdoba, Spain; q62regae@uco.es

**Keywords:** dispersive micro-solid phase extraction, magnetic polyamide nanocomposite, UPLC-DAD, benzophenones

## Abstract

In this article, the influence of the monomers on the extraction efficiency and the effect of the addition of surfactants during the synthesis have also been considered. The sorption capacity of the resulting nanocomposites has been evaluated, in the dispersive micro-solid phase extraction format, by determining that of six benzophenones in water using ultra performance liquid chromatography (UPLC) combined with photodiode array detection. Under the optimum conditions, the limits of detection were in the range of 0.5–4.3 ng/mL and the repeatability, expressed as the relative standard deviation (RSD), varied between 1.5% and 5.6%. The proposed method has been applied for the analysis of real water samples, providing relative recoveries in the interval of 84–105%

## 1. Introduction

Polymeric nanocomposites, obtained from the synergic combination of polymers and nanoparticles (NPs), have demonstrated great potential as sorbents in solid phase (micro)extraction [1,2]. In such a combination, polymers provide their well-established sorption capacity [3] while NPs boost the extraction capabilities (enhancing the superficial area of the polymer or introducing secondary interaction domains) [4,5,6] and endow the nanocomposite with unique properties [7,8]. The large number of available polymers (covering a wide range of different interaction chemistries) and NPs provide the analyst with an almost endless group of analytical tools that can be adapted to the problem (sample/analyte) under study. Polymeric nanocomposites have been traditionally obtained following three main routes, namely: (a) covering the polymer surface with the NPs; (b) coating the NP’s surface with the polymer; or (c) by electrospinning.

Covering the polymer surface with the NPs has been proposed as a synthetic approach, although it presents the reduction of the active surface of the polymer, due to the NPs’ deposition, as the main limitation [9]. The opposite approach has resulted in a better strategy since the resulting material maintains the nanometric size, which provides it with a high superficial area, and the polymer remains in the outer surface, making its interaction with the analytes possible [10,11]. However, this synthetic approach is usually multistep and is a relatively time-consuming procedure. On the other hand, electrospinning is a well-established and simple technique to obtain polymeric fibers with defined dimension and orientation. The introduction of NPs into the fiber mat is usually a priori done, dispersing the NPs into the precursor polymeric solution [12,13], although the later modification of the polymeric mate with NPs of a different nature, which are embedded in the network, is also possible [14].

In 2014, our research group proposed a new synthetic approach, which involves the embedding of the NPs into the polymeric network, to obtain nanocomposites simply and cheaply [15]. The procedure plays with the solvent-dependent solubility of polymers and consists of the solubilization of the polymer in an appropriate solvent, the dispersion of the NPs in this solution, and the final precipitation of the polymer around the NPs by a solvent changeover. The NPs interfere in the normal stacking of the polymer chains, increasing the superficial area of the polymer, [16] or they may provide new properties to the nanocomposite [17,18]. The synthesis can be considered environmentally friendly since the volume of organic solvent is reduced to 5 mL (formic acid) when polyamides are used. The scope of this strategy has been recently extended to other polymers, opening the door to the use of recyclable plastics in the preparation of nanocomposites [19].

Benzophenones are a family of organic UV-filters worldwide that are applied in many personal care products to protect our skin against damage by UVA and UVB radiation. They can also be found in some plastics, paints, adhesives, and rubber, where they are used to enhance the light stability of the materials. Due to their excessive use, they can find their way into wastewater, the environment, foods, and even the human body. There are increasing reports that BP-UV (benzophenone-UV) filters have the potential to interfere with the endocrine system [20,21]. Considering the adverse effects of long-term exposure and the excessive use of BP-UV filters, their identification in water samples is of great importance. This identification is usually done by liquid chromatography, coupled with different instrumental techniques. The low concentration of the targets in environmental waters makes a previous extraction to pre-concentrate them and to remove potential interferents necessary. Solid phase microextraction [22,23,24] and liquid phase microextraction [25,26,27,28] have already been proposed for this purpose. Dispersive micro-solid phase extraction, which is based on the efficient dispersion of a sorbent into the sample, is a powerful sample preparation technique that allows the rapid isolation of the target compounds [29]. In fact, some successful extraction protocols for the isolation of benzophenones have been reported [22,23]. In this article, an easy and fast (less than 5 h) procedure to synthesize polymeric nanocomposite, based on the use of Fe_2_CoO_4_ magnetic nanoparticles and polyamides, is proposed for the effective isolation of six benzophenones (BPs) from water samples. The influence of the type of polyamide used in the synthesis of the nanocomposite on the extraction efficiency of the nanocomposite is systematically evaluated. As a result, it has been demonstrated that the selection of the polyamide was critical in boosting the extraction of the analytes since the inclusion of aromatic rings on the structure allows better interaction with the targets. Moreover, the effect of the addition of a surfactant (SDS) as a potential modifier of the nanocomposite has been evaluated. In this case, a slightly higher porosity of the final nanocomposite was obtained. As a consequence, the enrichment factor was also higher for all of the analytes. Finally, the application of the synthesized material to the dispersive micro-solid phase extraction of the analytes resulted in detection limits in the low nanogram per liter range, with shorter extraction times.

## 2. Results and Discussion

**Figure 1:**
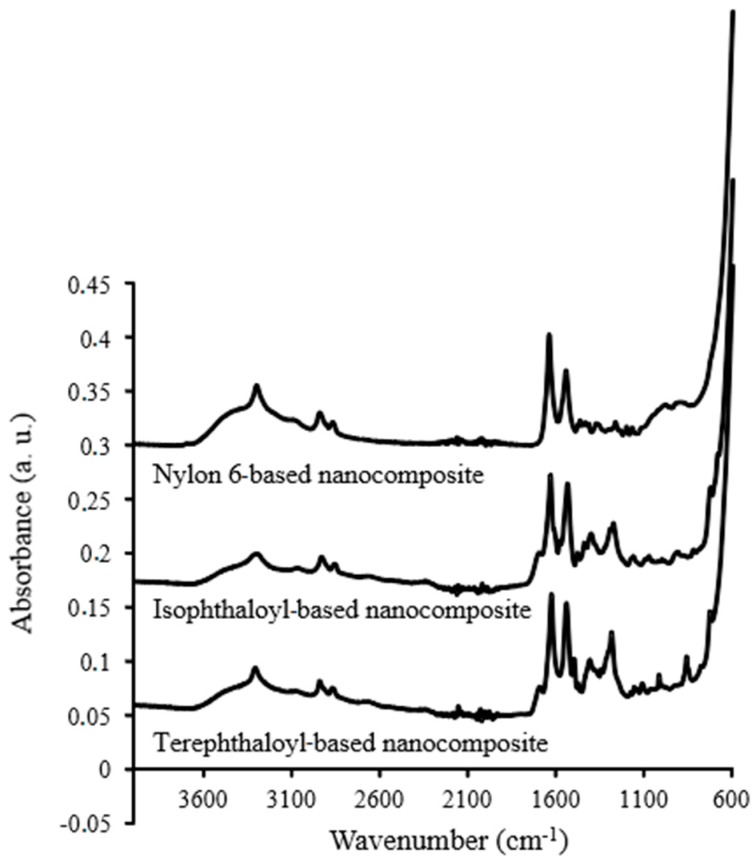
The IR (infrared) spectra of the resulting nanocomposites used in the study. The spectra are normalized to make their comparison easier.

The type of polyamide used in the nanocomposite synthesis is crucial since it defines the type of chemical interactions that the sorbent will establish with the target analytes [30]. Polyamides produce inter- and intra-molecular hydrogen bonds through their amide groups that allow the stacking of the linear polymer chains. These bonds may be developed with an external carbonyl, as are those presented in the benzophenone structures [31]. Also, the linear chains of polyamides can interact by dispersion forces with the analytes, while the introduction of aromatic moieties in the polymer can enhance the interaction with the benzene rings of the target compounds. Three different polyamides (commercial nylon-6 and lab-made isophthaloyl or terephthaloyl-based ones) were initially evaluated. The IR (infrared) spectra of the resulting nanocomposites are shown in Figure 1 show the characteristic bands of the polyamides. The band at around 1642 cm^−1^ is the result of the C=O stretching, while the band at around 1546 cm^−1^ is due to the N-H deformation.

**Figure 2:**
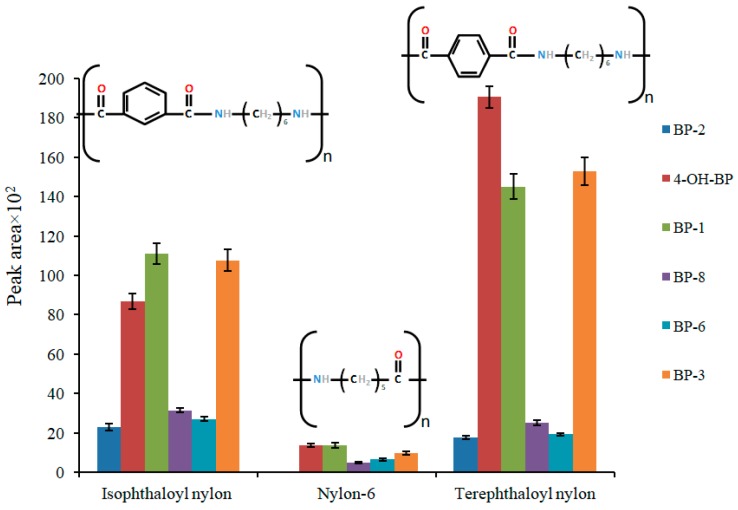
The effect of the polyamide type on the extraction of the target compounds. The chemical structure of the polyamides is also shown. Extraction conditions: Sample volume, 10 mL; sorbent amount, 20 mg; extraction time, 30 mins; sample pH, 7.4; elution solvent, methanol. The analytes are: 2,2′,4,4′-tetrahydroxybenzophenone (BP-2), 4-hydroxybenzophenone (4-OH-BP), 2,4-dihydroxybenzophenone (BP-1), 2,2′-dihydroxy-4-methoxybenzophenone (BP-8), 2,2′-dihydroxy-4,4′-dimethoxybenzophenone (BP-6), and 2-hydroxy-4-methoxybenzophenone (BP-3).

The extraction capabilities of the nanocomposites were evaluated, using aqueous standards containing the analytes at a concentration level of 100 ng/mL for each analyte. For this purpose, 10 mL of the standard was extracted with 20 mg of each composite during 30 min, with the analytes finally being eluted with 500 µL of methanol. In light of the results, which are shown in Figure 2, terephthaloyl polyamide-based nanocomposite performed the best. Nylon-6 provided the worst results, demonstrating the boosting effect of the inclusion of an aromatic moiety into the polyamide.

**Figure 3:**
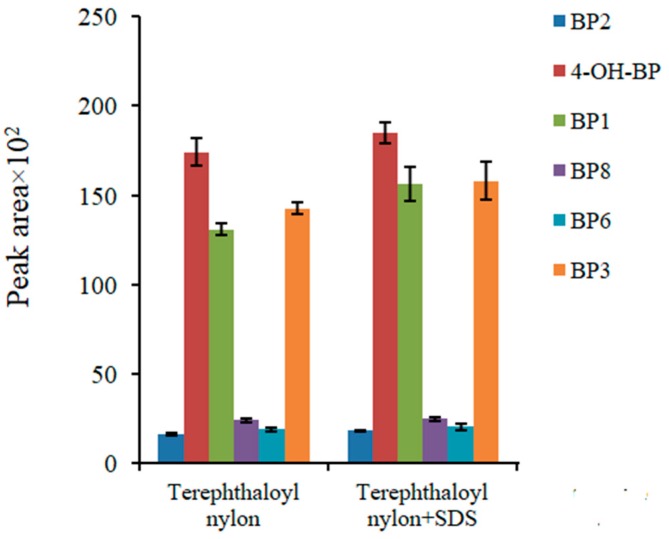
The effect of the addition of sodium dodecyl sulfate (SDS) on the extraction capabilities of the terephthaloyl-based magnetic nanocomposite. The analytes are: 2,2′,4,4′-tetrahydroxybenzophenone (BP-2), 4-hydroxybenzophenone (4-OH-BP), 2,4-dihydroxybenzophenone (BP-1), 2,2′-dihydroxy-4-methoxybenzophenone (BP-8), 2,2′-dihydroxy-4,4′-dimethoxybenzophenone (BP-6), and 2-hydroxy-4-methoxybenzophenone (BP-3).

The terephthaloyl polyamide-based nanocomposite produced a superficial area of 52 m^2^/g, which is an acceptable value for a solid phase extraction sorbent. The use of surfactants for increasing the superficial area has been successful for some nanoparticles [32]. In this case, the inclusion of SDS as a modifier only produces a slight improvement in the superficial area, to 57 m^2^/g. The results obtained, which are shown in Figure 3, also demonstrate a small increase in the extraction of the analytes.

### 2.1. Optimization of the Extraction Procedure

Several variables, listed in Table 1, were evaluated in the optimization of the extraction procedure. Table 1 also lists the initial values for these variables, the interval studied, and the optimum values achieved. For brevity, only those with a relevant effect on the extraction efficiency will be discussed.

**Figure 4:**
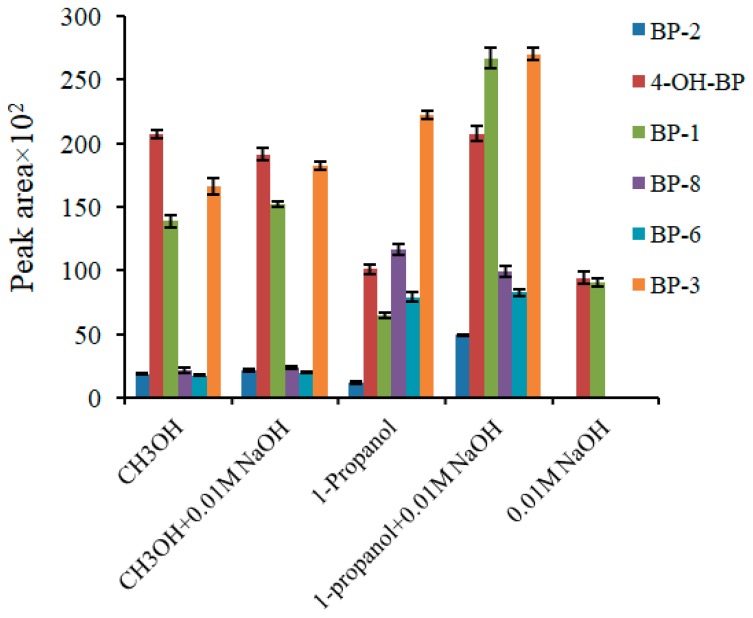
The effect of the elution solvent type on the efficiency of extraction. Extraction conditions: Sorbent, terephthaloyl nylon composite treated by sodium dodecyl sulfate (SDS); sample volume, 10 mL; sorbent amount, 20 mg; extraction time, 30 min; sample pH, 7.4. The analytes are: 2,2′,4,4′-tetrahydroxybenzophenone (BP-2), 4-hydroxybenzophenone (4-OH-BP), 2,4-dihydroxybenzophenone (BP-1), 2,2′-dihydroxy-4-methoxybenzophenone (BP-8), 2,2′-dihydroxy-4,4′-dimethoxybenzophenone (BP-6), and 2-hydroxy-4-methoxybenzophenone (BP-3).

Elution media is critical to achieving a good recovery of the extracted analytes from the sorbent. Considering the chemical nature of the analytes, four different media (methanol, 1-propanol, and 50/50 *v*/*v* mixtures of both solvents with 0.01 M NaOH aqueous solution) were tested. The results, summarized in Figure 4, showed that the 1-propanol mixed with 0.01 M NaOH solution provided the best results. 

**Figure 5:**
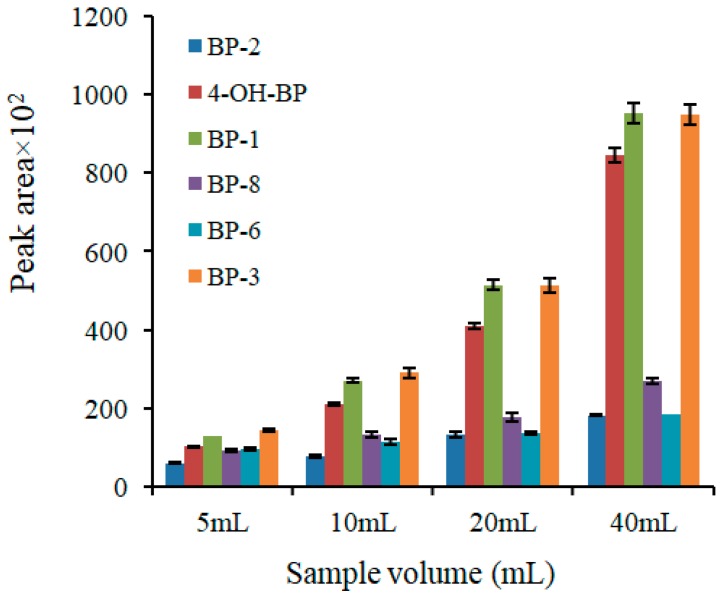
The effect of the sample volume on the efficiency of extraction. Extraction conditions: Elution solvent, mixture of 1-propanol and 0.01 M NaOH solution (50:50, *v*/*v*); extraction time, 30 min; sample pH, 7.4. The analytes are: 2,2′,4,4′-tetrahydroxybenzophenone (BP-2), 4-hydroxybenzophenone (4-OH-BP), 2,4-dihydroxybenzophenone (BP-1), 2,2′-dihydroxy-4-methoxybenzophenone (BP-8), 2,2′-dihydroxy-4,4′-dimethoxybenzophenone (BP-6), and 2-hydroxy-4-methoxybenzophenone (BP-3).

The sample volume was investigated in the range from 5 to 40 mL. As observed in our previous studies [17,19], the best dispersion of the nanocomposites was obtained when the sorbent/sample ratio was 2 mg per mL. This study was developed under these conditions. Thereby, the experiments were performed employing 10 to 80 mg of sorbent. As can be seen in Figure 5, the signals increased with an increasing sample volume. In light of these results, 40 mL was selected as the sample volume.

The extraction time was studied using an interval of 15 to 120 min. The extraction is generally fast and, in most cases, negligible variations were observed for the analytes. Therefore, the subsequent extractions were performed in 15 min, in order to maintain an acceptable sample throughput.

### 2.2. Analytical Parameters of the Method, Accuracy, and Real Sample Analysis

Once the optimum conditions were selected, the method was analytically evaluated in regards to the sensitivity, linear range, precision, and accuracy. The more relevant parameters are shown in Table 2. The calibration graphs were constructed by using aliquots of 40 mL of aqueous standards containing the benzophenones at concentrations between 0.5 and 5000 ng/mL and were processed following the complete dispersive micro-solid phase extraction procedure. Each standard was run in triplicate. The limits of detection (LOD) and limits of quantification (LOQ) were calculated based on an S/N of 3 and 10, respectively [33]. The LODs ranged from 0.5 to 4.3 ng/mL, while the LOQs (which were further defined as the initial points of the linear ranges) varied in the interval from 1.6 to 14.3 ng/mL. Good linearity (R^2^ ≥ 0.997) was observed for all of the analytes. The precision was evaluated using the repeatability and reproducibility at the concentration levels of 5 ng/mL for 4-OH-BP, BP-1, and BP-3; 12.5 ng/mL for BP-8 and BP-6, and 25 ng/mL for BP-2. The repeatability was evaluated by extracting six aliquots of a standard solution which provided values better than 5.6% (expressed as the relative standard deviation, RSD). The reproducibility was calculated by carrying out three replicate extractions in three subsequent days (*n* = 3). The values were better than 8.4%, expressed as the RSD. The enrichment factor (EF) values were calculated by comparison of the slopes of the calibration graphs obtained before and after the microextraction procedure. The EFs calculated thus varied between 6.1 to 24.1 and the absolute extraction recoveries were in the range of 7.6% to 30.1%.

In order to calculate the accuracy of the method, two methodologies can be followed. Firstly, a certified reference material (CRM) is the recommended approach. However, the availability of this material to the large variety of sample-analyte binomials is rather limited. Therefore, a second methodology is widely accepted in analytical sciences, which deals with the use of quality control samples (QC). This latter option has been selected in this article. For this purpose, validation samples were prepared using benzophenone-free water samples (from a tap, river, and creek). The samples were spiked with the analytes at 5 ng/mL for 4-OH-BP, BP-1, and BP-3; 12.5 ng/mL for BP-8 and BP-6; and 25 ng/mL for BP-2, before being analyzed. The samples were all run in triplicate and the relative recovery was calculated. An acceptable accuracy was defined as a relative recovery (RR) between 70–130%. As can be seen in Table 3, all of the values were between 84–105%, which indicates the absence of the matrix effect on the proposed dispersive micro-solid phase extraction method.

The present work was also compared with other works reported in the literature for the analysis of BPs [22,23,24,25,26,27,28]. Comparisons were performed regarding the LOD, linear dynamic range (LDR), intra-day RSD %, EF, relative recovery (RR %), sorbent synthesis time, and extraction time. Table 4 shows the results. As can be seen, better or comparable results were obtained by the present method in regards to the linearity, sensitivity, precision, and RR %. The main advantage of the current work is its simplicity and rapidity of the sorbent synthesis when it is compared with other sorbent-based methods. Additionally, it is completed in a short extraction time.

## 3. Material and Methods

### 3.1. Reagents

All of the reagents were of analytical grade or better. Unless otherwise specified, they were purchased from Sigma Aldrich (Madrid, Spain). 2,2′,4,4′-tetrahydroxybenzophenone (BP-2), 4-hydroxybenzophenone (4-OH-BP), 2,4-dihydroxybenzophenone (BP-1), 2,2′-dihydroxy-4-methoxybenzophenone (BP-8), 2,2′-dihydroxy-4,4′-dimethoxybenzophenone (BP-6), and 2-hydroxy-4-methoxybenzophenone (BP-3) were the target analytes. Stock standard solutions of the benzophenones were prepared at a concentration of 1000 mg/L in acetonitrile. The solutions were stored at 4 °C in the dark. Working solutions were prepared daily by the appropriate dilution of the stock solutions in methanol or Milli-Q water (Millipore Corp., Madrid, Spain), as required. Hexamethylendiamine, isophthaloyl chloride, terephthaloyl chloride, toluene, and methanol were used for synthesizing the different polyamides while commercial nylon-6 pellets were acquired. Sodium dodecyl sulfate (SDS) was evaluated as a modifier of the superficial area of the synthesized nanocomposites. 

Cobalt (II) nitrate hexahydrate, iron (III) nitrate nonahydrate, hydrogen peroxide (33.0 *w*/*v* %), and sodium hydroxide were used for the synthesis of the magnetic nanoparticles (Fe_2_CoO_4_). Formic acid and Milli-Q water were chosen as solvents, to induce the solubilization/precipitation of the polyamides around the magnetic core.

### 3.2. Preparation of the Magnetic Polyamide Nanocomposites (MNPs)

#### 3.2.1. Synthesis of the Magnetic Core (Fe_2_CoO_4_ MNPs)

Cobalt (II) nitrate hexahydrate (7.3 g) and iron (III) nitrate nonahydrate (20.2 g) were dissolved in 50 mL of Milli-Q water. The solution was heated at 80 °C and 250 mL of 1.2 M NaOH solution was added in a dropwise manner. The reaction proceeded at the same temperature for 2 h under vigorous stirring. Then, 250 mL of hydrogen peroxide (33.0 *w*/*v* %) was added in a dropwise manner and the dispersion was heated at 80 °C for 2 h. The resulting MNPs were isolated by an external magnet, washed (with Milli-Q water and acetonitrile), and finally dried in an oven at 80 °C.

#### 3.2.2. Synthesis of Isophthaloyl and Terephthaloyl Polyamides

Isophthaloyl and terephthaloyl polyamides were prepared according to our previous work [30]. Briefly, 4.06 g of isophthaloyl chloride or terephthaloyl chloride was dissolved in 20 mL of toluene. At the same time, 1.16 g of hexamethylenediamine and 0.3 g of NaOH were dissolved in 10 mL of water. Both solutions were transferred to a beaker, forming a biphasic system, with an interface at which the polyamide was formed. The resulting polyamide was collected by a glass rod, washed (with toluene, water, and methanol), and finally dried at 80 °C overnight.

#### 3.2.3. Synthesis of the Magnetic Polyamide Nanocomposites (MNPs)

For the preparation of the nanocomposites, 150 mg of polyamide (commercial nylon-6 or synthesized isophthaloyl or terephthaloyl polyamides) was dissolved in 5 mL of formic acid. 200 mg of MNPs were dispersed into the solution, aided by ultrasounds, for 5 min. Then, the dispersion was added to 50 mL of Milli-Q water using a plastic syringe. The solvent changeover, from formic acid to water, induced the precipitation of the polyamide around the MNPs. The resulting nanocomposite was recovered using an external magnet, was washed with water and acetonitrile, and was finally dried in the oven.

SDS was used as a potential modifier. Terephthaloyl polyamide nanocomposite was used in this study as it provided the best extraction results. For this purpose, 250 mg of the surfactant was added to the formic acid solution containing the polyamide and the MNPs, following a similar procedure to the one described above. The synthesis procedure was like the aforementioned but with minor changes. Once the nanocomposite was obtained, the modifier had to be removed with water.

### 3.3. Characterization of the Magnetic Polyamide Nanocomposites (MNPs)

The chemical composition of the synthesized nanocomposites was studied by infrared spectroscopy while their morphology was investigated using superficial area measurements.

Fourier transform infrared (FT-IR) spectra were obtained with a Bruker Tensor 37 FT-IR spectrometer, equipped with a Deuterated Triglycine Sulfate (DTGS) detector. The spectrometer consisted of a diamond ATR cell with a circular surface of 3 mm in diameter and three internal reflections. The spectra were recorded in the range of 600–4000 cm^−1^ at a 4 cm^−1^ resolution with 64 co-added scans. The data acquisition was performed by OPUS software (version 4.2, Bruker, Ettligen, Germany).

The measurements of the superficial areas of the composites were carried out using a Quanta chrome^®^ ASiQwinTM-Automated Gas Sorption Data analyzer (Quantachrome, Boynton Beach, FL, USA) at the Institute of Fine Chemistry and Nanochemistry at the University of Cordoba. The analysis was based on the nitrogen adsorption/desorption at −196 °C and the specific surface area values were calculated using the BET (Brunauer–Emmett–Teller) equation. 

### 3.4. Extraction Procedure

**Figure 6:**
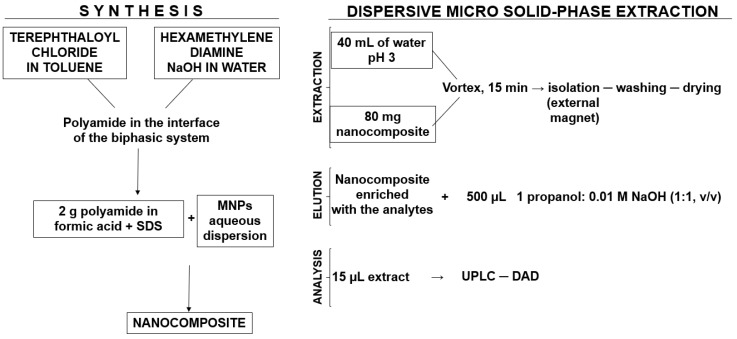
The scheme of the synthesis of the nanocomposite and its use in the dispersive micro-solid phase extraction procedure designed for the identification of benzophenones in waters. SDS: Sodium dodecyl sulfate; MNPs: Magnetic Polyamide Nanocomposites; UPLC-DAD: Ultra-Performance Liquid Chromatography with Diode-Array Detection.

Firstly, 80 mg of the nanocomposite was placed in a 50 mL vial and 40 mL of an aqueous standard or a water sample, adjusted at pH 3 with a diluted hydrochloric acid solution, was poured into the vial. The vial was vortexed vigorously for 15 min. The use of a multiport vortex allowed the simultaneous extraction of several samples. The sorbent was isolated from the solution by an external magnet and washed with 10 mL of Milli-Q water. After that, the sorbent was completely dried and the analytes were eluted by 500 µL of a mixture of 1-propanol and 0.01 M NaOH solution (50:50, *v*/*v*). The eluate was finally analyzed by Ultra-Performance Liquid Chromatography with Diode-Array Detection (UPLC-DAD) (Waters Corp., Madrid, Spain). The synthesis pathway, as well as the dispersive microextraction procedure, is depicted in Figure 6.

### 3.5. UPLC (Ultra-Performance Liquid Chromatography) Analysis

The analytes were separated and quantified using a Waters Acquity TM Ultra Performance LC system (Waters Corp., Madrid, Spain) equipped with an autosampler and a PDA eλ Detector (Waters). The analytical column was an ACQUITY UPLC^®^BEH C18 column (1.7 µm, 2.1 × 100 mm) that was maintained at 30 °C. The separation was performed under a gradient elution mode using a mobile phase consisting of water (solvent A) and acetonitrile (solvent B). The elution began by using 20% of solvent B and linearly increased to using 60% within 8 min. The flow rate was maintained at 0.5 mL/min and the injection volume was 5 μL. The detection was performed at 290 nm.

## 4. Conclusions

In this article, we have presented the synthesis of a nanocomposite for the identification of benzophenones in water samples. The sorbent was laboratory-prepared by the simple mixing of the components. In the first stage, the polymeric phase was synthesized using monomers whose chemical structure maximized the interaction with the analytes. In this case, the presence of aromatic rings is crucial. Next, and with the aim of increasing the porosity, two strategies were implemented. Magnetic nanoparticles were incorporated into the polymeric network by playing with the solubility of the polymer in different media. In this way, the addition of an aqueous dispersion of the MNPs to a solution of the polymer in formic acid immediately precipitated the nanocomposite. The inclusion of the MNPs between the polymer chain increased the porosity of the material. Also, the presence of the surfactant SDS in the formic phase permitted the incorporation of the micelles into the polymeric network. Washing the solid with water removed the suprastructures, leaving the voids within the nanocomposite. This resulted in a higher porosity in comparison to the material prepared in the absence of SDS. The sorbent has been used in a dispersive micro-solid phase extraction procedure, where the superparamagnetism exhibited by the solid simplifies the whole methodology, as no filtration/centrifugation steps are required. Rather, an external magnet enabled the recovery of the nanocomposite in different stages of the procedure. The developed protocol provided enrichment factors of between 6.1–24.1. These values are acceptable, but they can be improved eventually (by up to 10-times) if an evaporation/redissolution of the final methanolic extract is implemented prior to the chromatographic analysis.

## Figures and Tables

**Table 1:** A list of the variables studied in extraction optimization.

Variable	Initial Value	Interval Studied	Optimum Value
Elution solvent	Methanol	Several solvents	Mixture of 1-propanol and 0.01 M NaOH solution (50:50, *v*/*v*)
Elution temperature (°C)	RT ^1^	RT ^1^/60	RT ^1^
Sample volume (mL)	10	5–40	40
Extraction time (min)	30	15–120	15
pH	7.4	3–9	3

^1^ RT, room temperature.

**Table 2:** The analytical parameters of the proposed method for the selected benzophenones (BPs).

Analyte	Linear Range (ng/mL)	Determination Coefficient (R^2^)	LOD ^a^ (ng/mL)	AER ^b^ (%)	EF ^c^	Intra-Day RSD % (*n* = 6) ^d^	Inter-Day RSD % (*n* = 3) ^d^
BP-2	14.3–5000	0.997	4.3	7.6	6.1	5.6	8.0
4-OH-BP	2.0–1000	0.997	0.6	20.6	16.5	1.9	3.8
BP-1	1.6–1000	0.998	0.5	28.8	23.0	1.5	4.6
BP-8	7.2–2500	0.999	2.2	8.9	7.1	1.7	7.7
BP-6	10–2500	0.998	3.0	9.0	7.2	4.9	5.3
BP-3	1.7–1000	0.997	0.5	30.1	24.1	3.0	8.4

^a^ Limit of detection; ^b^ Absolute extraction recovery; ^c^ Enrichment factor; ^d^ Relative standard deviation. The analytes are: 2,2′,4,4′-tetrahydroxybenzophenone (BP-2), 4-hydroxybenzophenone (4-OH-BP), 2,4-dihydroxybenzophenone (BP-1), 2,2′-dihydroxy-4-methoxybenzophenone (BP-8), 2,2′-dihydroxy-4,4′-dimethoxybenzophenone (BP-6), and 2-hydroxy-4-methoxybenzophenone (BP-3).

**Table 3:** The analysis of water samples spiked with the target analytes.

Analyte	Creek (RR ± SD) ^a^	Tap (RR ± SD) ^a^	River (RR ± SD) ^a^
BP-2	84 ± 3	87 ± 3	105 ± 4
4-OH-BP	96 ± 3	98 ± 2	86 ± 3
BP-1	100 ± 2	100 ± 3	96 ± 1
BP-8	96 ± 5	104 ± 5	102 ± 3
BP-6	90 ± 2	103 ± 4	104 ± 4
BP-3	98 ± 3	97 ± 2	97 ± 2

^a^ Relative recovery percentage ± standard deviation (*n* = 3). The analytes are: 2,2′,4,4′-tetrahydroxybenzophenone (BP-2), 4-hydroxybenzophenone (4-OH-BP), 2,4-dihydroxybenzophenone (BP-1), 2,2′-dihydroxy-4-methoxybenzophenone (BP-8), 2,2′-dihydroxy-4,4′-dimethoxybenzophenone (BP-6), and 2-hydroxy-4-methoxybenzophenone (BP-3).

**Table 4:** A comparison of the proposed method with other reported methods.

Method	Analyte	LOD (ng/mL)	LDR (ng/mL)	Intra-Day RSD %	EF	RR %	Synthesis Time (hours:mins)	Extraction Time (mins)	Reference
Magnetic D-µ-SPE-UPLC-DAD	BP-2	4.3	14.3–5000	5.6	6.1	84–105	4:45	15	Present method
	4-OH-BP	0.6	2–1000	1.9	16.5	86–98			
	BP-1	0.5	1.6–1000	1.5	23	96–100			
	BP-8	2.2	7.2–2500	1.7	7.1	96–104			
	BP-6	3.0	10–2500	4.9	7.2	90–104			
	BP-3	0.5	1.7–1000	3.0	24.1	97–98			
D-µ-SPE-HPLC-DAD	BP-1	1.2	4–3500	1.5	-	-	37:00	55	[22]
	BP-3	0.9	4–3500	2.3	-	-			
Magnetic D-µ-SPE-HPLC-MS/MS	BP-2	0.81	2.7–500	5.2	21.4	92–96	132:30	27	[23]
	4-OH-BP	0.62	2.07–500	4.2	29.3	90–93			
	BP-1	1.21	4.03–500	7.1	17.3	89–92			
	BP-8	0.84	2.80–500	5.1	34.1	87–96			
	BP-6	1.11	3.70–500	8.3	18.4	92–98			
	BP-3	0.16	0.87–500	7.3	49.2	89–96			
BAµE-HPLC-DAD	4-OH-BP	Sorbent 1: 0.3	1–24	5.6	-	-	-	Sorbent 1: 255 Sorbent 2: 990	[24]
		Sorbent 2: 0.4	2–24	3.4	-	-			
	BP-1	Sorbent 1: 0.3	1–24	5.0	-	-			
		Sorbent 2: 0.4	2–24	1.5	-	-			
	BP-3	Sorbent 1: 0.3	1–24	2.6	-	-			
		Sorbent 2: 0.4	2–24	8.5	-	-			
MR-DLLME-HPLC-DAD	4-OH-BP	0.7	70–7000	4.1	93	-	28:40	5	[25]
	BP-1	1.8	70–7000	3.7	110	-			
	BP-3	12.3	70–7000	4.4	107	-			
IL-HF-LPME-HPLC-UV	BP-1	0.5	10–1000	8.2	25	101.9–102.7	-	50	[26]
	BP-3	0.2	5–1000	1.1	216	98.1–104.9			
IL-DLLME-HPLC-UV	4-OH-BP	2.4	10–1000	6.3	21.7	-	-	14	[27]
	BP-1	6.4	20–1000	4.1	18.9	-			
	BP-3	3.3	10–1000	8.0	20.3	-			
USA-DLLME HPLC-UV	BP	0.15	0.5–500	4.0–5.9	76	92–102	-	9	[28]
	BP-1	0.15	0.5–500	2.4–4.2	67	90–103			
	BP-3	0.30	1–500	3.7–5.1	74	94–100			

SPE, solid phase extraction; DAD, diode array detection; BAµE, bar adsorptive micro-extraction; MR-DLLME, magnetic retrieval dispersive liquid-liquid microextraction; IL-HF-LPME, Ionic liquid Hollow fiber liquid phase microextraction; USA, ultrasound assisted. The analytes are: 2,2′,4,4′-tetrahydroxybenzophenone (BP-2), 4-hydroxybenzophenone (4-OH-BP), 2,4-dihydroxybenzophenone (BP-1), 2,2′-dihydroxy-4-methoxybenzophenone (BP-8), 2,2′-dihydroxy-4,4′-dimethoxybenzophenone (BP-6), and 2-hydroxy-4-methoxybenzophenone (BP-3). LOD, limit of detection; LDR, linear dynamic range; RSD, relative standard deviation; EF, enrichment factor; RR, relative recovery.
